# The Executive Committee of the American Dental Society of Europe

**Published:** 1897-08

**Authors:** L. A. O’Brian

**Affiliations:** 15 Walpurgisstrasse, Dresden


### The Executive Committee of the American Dental Society of Europe.

The Executive Committee of the American Dental Society of Europe has decided to postpone the meeting arranged for this year in London, until August, 1898.

The next meeting will celebrate the 25th anniversary of the founding of this Society and it is hoped that all members will participate in making it a brilliant success.

By order of the Executive Committee.

L. A. O'BRIAN, *Secretary*,
15 Walpurgisstrasse, Dresden.

